# Determinants of hypertensive crisis among hypertensive patients at adult emergency departments of public hospitals in eastern Ethiopia, 2023: A case-control study

**DOI:** 10.1371/journal.pone.0326055

**Published:** 2025-06-16

**Authors:** Fenta Wondimneh, Melaku Getachew, Tilahun Teshager, Henok Legesse, Ayichew Alemu, Indeshaw Ketema, Dejene Tesfaye, Yalew Mossie, Natan Muluberhan, Fentahun Meseret

**Affiliations:** 1 Department of Emergency and Critical Care Nursing, College of Health and Medical Sciences, Haramaya University, Harar, Ethiopia; 2 Department of Emergency and Critical Care Medicine, School of Medicine, College of Health and Medical Sciences, Haramaya University, Harar, Ethiopia; 3 School of Nursing, College of Health and Medical Sciences, Haramaya University, Harar, Ethiopia; 4 Department of Pediatrics and Child Health Nursing, School of Nursing, College of Health and Medical Sciences, Haramaya University, Harar, Ethiopia,; 5 Department of psychiatry, College of Health and Medical Sciences, Haramaya University, Harar, Ethiopia; Christian Medical College, INDIA

## Abstract

**Background:**

Hypertensive crisis is a life-threatening condition requiring urgent medical intervention. Identifying key determinants is essential for effective prevention and management. This study aimed to assess factors associated with hypertensive crisis among patients attending selected public hospitals in eastern Ethiopia.

**Methods:**

A hospital-based unmatched case-control study was conducted among 357 participants (119 cases and 238 controls). Cases were hypertensive crisis patients, while controls were hypertensive patients without crisis in adult emergency departments of public hospitals in eastern Ethiopia from April 10th to September 10th, 2023. Data were collected using structured questionnaires and medical record reviews. Multivariable logistic regression was used to identify independent determinants of hypertensive crisis, with results presented as Adjusted Odds Ratios (AORs) and 95% Confidence Intervals (CIs).

**Results:**

The mean age of participants was 54.7 ± 14.4 years for cases and 48.14 ± 15.51 years for controls. More than half of the cases (54.8%) and one-third of the controls (32.6%) were female. Multivariable analysis identified female sex (AOR = 2.91, 95% CI: 1.67–5.07), unemployment (AOR = 2.28, 95% CI: 1.20–4.33), diabetes mellitus (AOR = 3.33, 95% CI: 1.59–7.01), previous history of hypertension (AOR = 2.25, 95% CI: 1.21–4.19), drinking alcohol (AOR = 3.01, 95% CI: 1.45–6.24), and poor knowledge of hypertension (AOR = 1.86, 95% CI: 1.07–3.22) as significant determinants of hypertensive crisis (p < 0.05).

**Conclusion:**

Female sex, unemployment, diabetes mellitus, history of hypertension, drinking alcohol, and poor hypertension knowledge were independent predictors of hypertensive crisis. However, the case-control design’s intrinsic limitations make it difficult to establish a temporal relationship between risk factors and outcomes. Strengthening hypertension education, promoting lifestyle modifications, and enhancing healthcare access may help mitigate the risk of hypertensive crisis.

## Introduction

Hypertension (HTN), a common health problem worldwide, is diagnosed through two or three evaluations spaced by 1–4 weeks apart if the systolic blood pressure (SBP) is ≥ 140 mm Hg and/or the diastolic blood pressure (DBP) is ≥ 90 mm Hg [1]. If there is evidence of organ damage and the blood pressure is greater than 180/110 mm Hg, it can be detected with just one measurement [1]. HTN is primarily defined as elevated arterial blood pressure and is the leading cause of morbidity and mortality in both high- and low-income countries [2]. According to a systematic review and meta-analysis, Ethiopia has a pooled HTN prevalence of 20.63% [3]. The prevalence is particularly high in Dire Dawa City Administration and Jigjiga City in the Somali Regional State of eastern Ethiopia, with rates of 24.43% and 28.3%, respectively [4,5].

Hypertensive crisis (HTN-crisis) is considered as severe form of hypertension, and described as a systolic blood pressure elevation greater than or equal to 180 mmHg and/or a diastolic blood pressure elevation greater or equal to 120 mmHg [6]. HTN-crisis could be classified as either hypertensive emergency (HTN-emergency) or hypertensive urgency (HTN-urgency) depending on target organ damage is present or absent, respectively [7]. HTN-crisis makes up around 1–2% of the global prevalence of HTN, and it makes up 1–15% of recognized hypertensive patients who have received prior treatment [2,8]. According to a study in Tanzania, approximately 68% of hypertension patients admitted to the emergency room had a HTN-emergency [9].

Globally, HTN-crises can cause significant morbidity and mortality; the annual death rate from HTN emergencies is over 79%, and the median survival period is 10.4 months if treatment is not appropriately administered [10]. Studies show that the effects of HTN-crises on different organs account for 36% of cardiovascular conditions, 24% of cerebral infarction, 16% of hypertensive encephalopathy, 12% of acute coronary syndrome (ACS), 4% of intracerebral or subarachnoid hemorrhage, 2% of patients with aortic dissection, and 5% of eclampsia during pregnancy [11].

Depending on which organs are damaged, patients with a HTN-crisis may experience breathing difficulties, headaches, dizziness, flank pain, oliguria, bloody urine, blurred vision, nausea, vomiting, palpitations, altered mental status, and other symptoms [12,13]. The clinical manifestations of HTN-crisis vary across patients based on the extent of organ damage, and can even occur in the absence of organ impairment [7,14].

HTN-crisis are primarily linked to stopping antihypertensive medication abruptly or not taking it as prescribed [15]. Other risk factors include drug and substance abuse, vascular diseases, brain injury, female gender, high body mass index, cardiac illness, and mental illness [16,17]. Additionally, it may be associated with psychological and emotional stress, improper salt and fluid intake, excessive alcohol use, a history of preeclampsia in women, and endocrine problems, including diabetes mellitus [8,11].

Despite the lack of research on HTN-crisis in Ethiopia, 523 patients with the diagnosis of HTN-crisis visited Gondar University Hospital between 2013 and 2016; 159 patients with the same diagnosis visited Ayder Specialty Hospital between 2018 and 2019 [18,19]. Even so, very little research has been done in sub-Saharan Africa including Ethiopia, and none has been done on its determinants in the area of interest. Therefore, the aim of this study was to assess determinants of hypertensive crisis among hypertensive patients at adult emergency departments (ED) of public hospitals in eastern Ethiopia.

## Methods and materials

### Study settings and duration

The study was conducted in selected four public hospitals of eastern Ethiopia from April 10th to September 10th, 2023. The study encompasses high emergency patient flow public hospitals in Harari Regional State, Somali Regional State, Dire Dawa City Administrations and Eastern Harrarge zone of Oromia Regional State. Harar is the capital city of Harari Regional State, which is 526 km toward east of Addis Ababa, the capital city of Ethiopia. According to central statistical agency population projection by 2022, the region had an estimated population of 276,431 [20]. The region has 2 public hospitals (Hiwot Fana Comprehensive Specialized Hospital (HFCSH) and Jugol General Hospital). HFCSH was selected for the high emergency patient visits.

Dire Dawa, a self-administrative city, is located 515 km toward east of Addis Ababa. The administration had an estimated population projection of 535,684 by 2022 [20]. Dire Dawa has 2 public hospitals (Dilchora Referral Hospital (DRH) and Sabian General Hospital). DRH was selected due to its high emergency patient flows.

Jigjiga, the capital city of Somalia Regional State, is located 625 km from Addis Ababa and 100 km from Harar in the eastern part of Ethiopia. The regional state had an estimated population projection of 6,506,235 by 2022 [20]. Jigjiga University Sheik Hassan Yabare Referral Hospital (JUSHYRH) was selected for high patient visits.

Haramaya district is one of the 22 district of East Hararghe zone, Oromia Regional State. According to the 2007 national census, the total population of the district is 271,018 [21]. Haramaya General Hospital (HGH) is the only public hospital in this district and selected for this study.

### Study design and populations

A hospital based unmatched case-control study was conducted. Adult hypertensive patients with SBP ≥ 180 mmHg and/or DBP ≥ 120 mmHg, and those with SBP ≥ 140 mmHg and/or DBP of ≥90 mmHg who visited the adult ED of selected public hospitals were considered cases and controls, respectively. All adult hypertensive patients who visited the adult ED of selected public hospitals in eastern Ethiopia during the study period were the study populations. Hypertensive patients aged less than 18 years, patients with cognitive impairment, seriously ill patients who were unable to finish the interviews, and patients with incomplete medical records were excluded from this study.

### Sample size determination

The sample size was determined based on factors significantly associated with the outcome variable in a previous study. Using Epi info version 7.0 and the double population proportion formula, the required sample size was calculated with the following parameters: 95% significance level, 90% power, Adjusted Odds Ratio (AOR) = 2.494, and a 1:2 case-to-control ratio with a 5% margin of error. The percentage of cases among female patients (P1) and the percentage of controls exposed (P2) were 36.4% and 63.6%, respectively. The odds ratio was derived from a study conducted in Mekelle, North Ethiopia, which identified female sex as a risk factor for hypertensive crisis [18]. To obtain the maximum sample size, p2 and the corresponding adjusted odds ratio were used. Then the total calculated sample was 324 (108 cases and 216 controls). To account for potential non-responses, we assumed a 10% non-response rate. This led to an adjusted sample size of 119 cases and 238 controls, resulting in a final total sample size of 357 participants.

### Sampling techniques and procedures

Based on the information obtained from the hospital’s Health Management Information System (HMIS) or ED registry, 174 HTN- crisis cases were visited ED of the four public hospitals (HFCSH = 48, DRH = 42, HGH = 30, HYJUH = 54) from April 10 to September 10, 2022. Depending on this baseline data, an estimated number of study participants were proportionally allocated to respective hospitals using probability proportional to size formula (HFCSH = 33, DRH = 29, HGH = 20, and HYJUH = 37). For each case, the data collectors took two consecutive controls. All eligible patients were interviewed until the required sample size was obtained.

### Data collection tools and methods

Data were collected using a pretested and structured questionnaire adapted from relevant literatures [18,19,22–25]. To ensure the validity of the data collection tool, the questionnaire was initially developed in English, translated into local languages (Afan Oromo, Amharic and Afsomali), and subsequently back-translated into English by language experts to verify consistency and accuracy. The questionnaire was comprised of four parts; Socio-demographic characteristics (8 items); vital signs (3 items); comorbidities (11 items); and other determinants (medication adherence, patients knowledge of hypertension, and personal behavior related determinants) contains 29 items.

Data were collected through a face-to-face interview and medical record review over a period of 6 months. First, a brief orientation was given to data collectors on the study objectives, sampling, data clarity and completeness. It is collected by 16 trained BSc nurses (four for each hospital) and 4 master of emergency and critical care nurse professionals as supervisors (one for each hospital). Next, data collectors described the objectives of the study and sought informed, written and signed consent from the study participants. Then finally, all cases and two consecutive controls who visited the selected hospitals during the study period were interviewed until the calculated sample is obtained. Data regarding patient’s diagnoses, vital signs and comorbidities were taken from their medical records.

To assess participants’ adherence to antihypertensive medications, we used the Morisky Medication Adherence Scale (MMAS-8), which consists of eight items. For the first seven items, responses were scored as “yes” = 1 and “no” = 0. The final item used a five-point Likert scale with the options “never,” “once,” “sometimes,” “usually,” and “always.” Adherence levels were categorized based on the total score: a score of less than 6 indicated low adherence, scores between 6 and 8 were considered medium adherence, and a score of 8 reflected high adherence [22].

Participants’ knowledge of hypertension was evaluated using a 14-item tool adapted from a previous study. Scores below the mean were classified as indicating ‘poor knowledge,’ while those at or above the mean were classified as having ‘good knowledge’ [26].

### Variables

The dependent variable for this study was hypertensive crisis. Independent variables include socio-demographic factors (age in years, sex, residency, educational status, and marital status), knowledge HTN, adherence to antihypertensive medications, comorbidities (diabetes mellitus (DM), stroke, acute coronary syndrome, congestive heart failure, chronic renal disease), behavioral and other potential determinants (Previous history of HTN, Family history of HTN, regular follow up, started taking antihypertensive medications, cigarette smoking, khat chewing, and alcohol use).

### Operational definitions

**Hypertensive crisis:** a severe form of hypertension when SBP is ≥ 180 and or DBP is ≥ 120 mmHg with or without evidence of organ damage [6,27].

**Co-morbidities:** respondents with one or more medical conditions in addition to hypertensions [28].

**Level of medication adherence**: Defined as low, medium, or high adherence to antihypertensive medications based on the Morisky Medication Adherence Scale (MMAS-8). Patients scoring < 6 were considered to have low adherence; scores between 6 and 8 indicated medium adherence, and a score 8 was classified as high adherence [22,29].

**Started taking antihypertensive medications:** A patient starts anti-hypertensive medications after meeting the national protocol of medication initiation; a patient taking any of mono or combinations of antihypertensive medications were considered as started medication.

**Patient knowledge about hypertension:** Defined as the level of knowledge of hypertensive patients about hypertension, assessed using a 14 item assessment tool. Participants scoring below the mean were considered as have poor knowledge, and those scoring at or above the mean were classified as having good knowledge [26].

### Data quality control

The questionnaire was checked for its coherence & completeness. A pretest was done among 10% of the total sample size at two none selected hospitals (Jugol General Hospital and Bisidimo Hospital) to enhance the reliability of instrument. Training was given for data collectors, the data clerk, and the supervisor prior to the actual data collection. The principal investigator and supervisor made spot-checking and reviewing the completed questionnaires to ensure completeness and consistency of the information collected.

### Data processing and analysis

The collected data were cleaned for completeness and consistencies before data entry. Responses to each question were coded for simplicity of data entry. The coded data were entered in to Epi data 4.2.0 and exported to SPSS version 26.0 statistical software for data analysis. In the first step the descriptive analysis like percentages, frequency distribution and measures of central tendency were computed. Binary logistic regression was done and variables with a p-value less than 0.25 were eligible for the final model. A multivariable logistic regression was performed to identify the independent predictors of HTN-crisis. A statistical significant level was declared at a p-value of less than 0.05. Hosmer-Lemeshow goodness of fit statistics and the model was adequately fitted with a p-value > 0.05.

### Ethics approval and consent to participate

The research was conducted according to the recommendations of the declarations of Helsinki [30]. Ethical approval was obtained from the Institutional Health Research Ethics Review Committee (IHRERC) of Haramaya University College of Health and Medical Sciences on 03/21/2023. (Ref No. IHRERC/048/2023). A formal letter of ethical approval obtained from the IHRERC was sent to selected public hospitals to obtain administrative permission. Additionally, permission letters from the selected public hospitals were obtained. Informed verbal and written consent was obtained from study participants. The participant’s identity was kept secret during data collection and dissemination processes.

## Results

### Sociodemographic characteristics of the respondents

The final sample size for this study was 357; however, due to participant refusals, only 345 participants (115 cases and 230 controls) completed the study, resulting in a response rate of 96.63%. The mean age was 54.7 ± 14.4 years for cases and 48.14 ± 15.51 years for controls. Nearly half of the cases (46.1%) and 37.8% of controls were aged 46–65 years. Females comprised 54.8% of cases and 32.6% controls. More than half of the cases (53%) and controls (64.3%) were Muslim religion followers. Urban residents comprised 57.4% of cases and 55.7% of controls. Regarding education level, the majority of both cases (71.3%) and controls (85.2%) had an education level of secondary school or lower, while fewer participants had a college-level education or higher (28.7% of cases and 14.8% of controls). As for employment status, most of the cases (68.7%) and controls (81.3%) were employed, whereas a higher percentage of cases (31.3%) were unemployed compared to the controls (18.7%). Most participants were married (67.8% of cases, 80.9% of controls). In terms of study participants’ monthly income, 13% of the cases and 11% of the controls earned less than 1000 Ethiopian Birr (ETB). (Table 1).

**Table 1:** Sociodemographic characteristics of the respondents at selected public hospitals of eastern Ethiopia, 2024.

Variables	Category	Group of patients (N = 345)	
		Cases, n (%)	Controls, n (%)
Age category	18-45	38(33.0)	109(47.4)
	46-65	53(46.1)	87(37.8)
	>65	20(20.9)	34(14.8)
Sex	Male	52(45.2)	155(67.4)
	Female	63(54.8)	75(32.6)
Religion	Orthodox	43(37.4)	59(25.7)
	Muslim	61(53.0)	148(64.3)
	Protestant	9(7.8)	19(8.3)
	Catholic	4(1.7)	4(1.7)
Residency	Rural	49(42.6)	102(44.3)
	Urban	66(57.4)	128(55.7)
Educational status	No formal education	16(13.9)	25(10.9)
	Primary (1-8)	19(16.5)	34(14.8)
	Secondary (9-12)	47(40.9)	137(59.6)
	Collage & above	33(28.7)	34(14.8)
Employment	Unemployed	36(31.3)	43(18.7)
	Employed	79(68.7)	187(81.3)
Marital status	Single	37(32.2)	44(19.1)
	Married	78(67.8)	186(80.9)
Monthly income in ETB	<1000	15(13.0)	25(10.9)
	1000-5000	33(28.7)	77(33.5)
	>5000	67(58.3)	128(55.7)

The mean systolic blood pressure (SBP) and diastolic blood pressure (DBP) values were significantly higher among cases compared to controls. Specifically, cases had a mean SBP of 208.2 ± 16.1 mmHg and a mean DBP of 129.3 ± 15.4 mmHg, whereas controls had a mean SBP of 161.3 ± 10.7 mmHg and a mean DBP of 100.1 ± 6.5 mmHg.

### Participants’ comorbidity conditions

**Fig 1:**
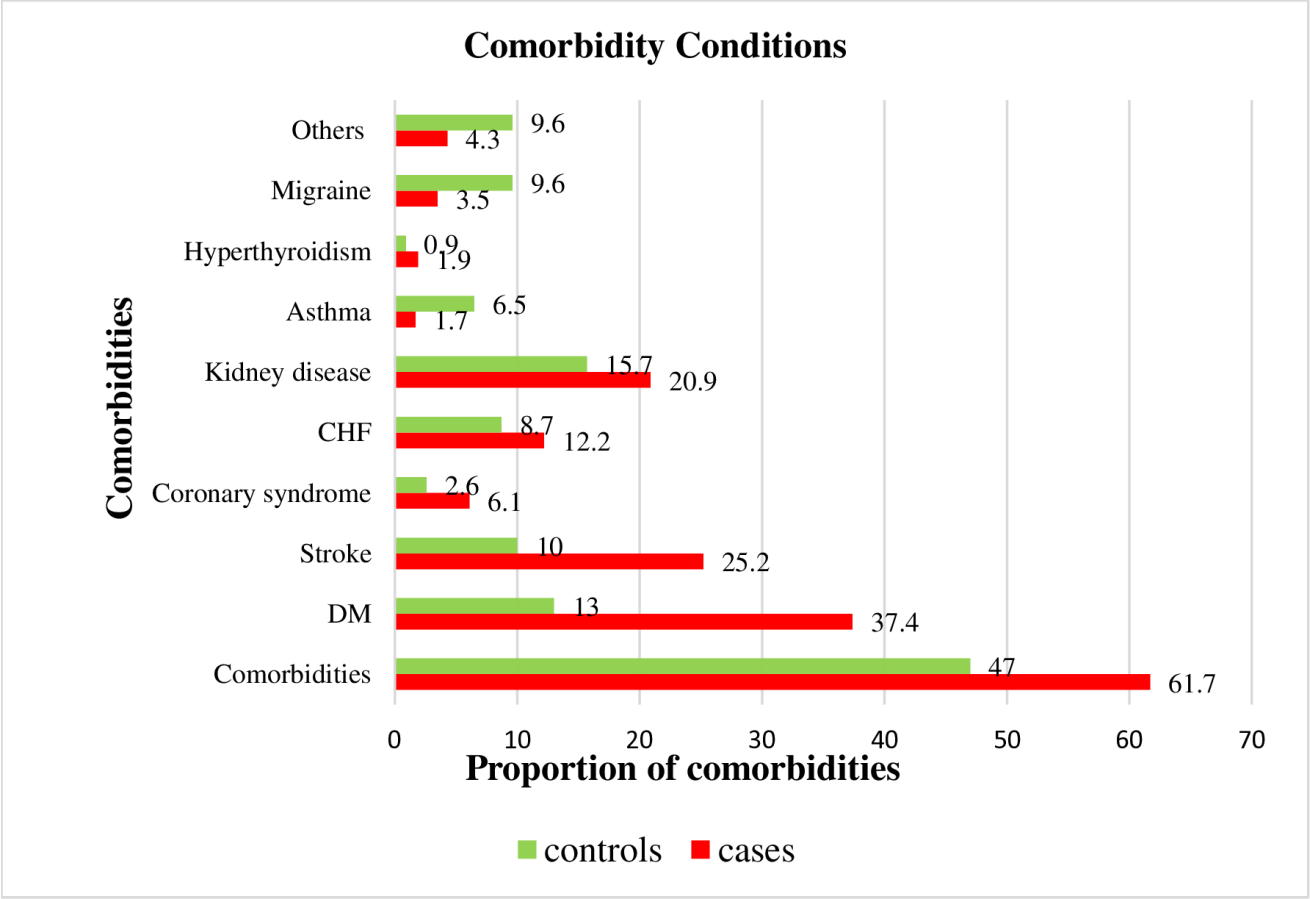
Comorbidity determinants of hypertensive crisis among respondents at selected public hospitals of eastern Ethiopia, 2024.

More than half of the cases (61.7%), and nearly half of the controls (47.0%) had comorbidities. Diabetes mellitus (DM) and stroke were more common among cases than controls (37.4% vs 13.0% and 25.2% vs 10.0% respectively). Similarly, congestive heart failure (CHF) (12.2% vs 8.7%) and kidney disease (20.9% vs 15.7%) were also more prevalent among cases (Fig 1).

### Behavioral and other determinants

A previous history of HTN was more common among cases (80%) than controls (57%). More than half of both groups had started antihypertensive medications (60% of cases and 53.9% of controls). Regular follow-up was low, with only 35.7% of cases and 33.9% of controls attending consistently. The proportion of respondents with family history of HTN among cases and controls was almost similar(47.8% vs 47.4%). Cigarette smoking was more prevalent among cases than controls (56.5% vs 37.8%). In contrast, Khat chewing was slightly lower (64.3% vs 69.1%). Alcohol drinking was higher among cases (28.7%) than controls (11.3%).

In this study, the proportion of good knowledge of HTN was lower among cases (40.9%) than control groups (60.9%). Approximately equal proportions of both groups had a high level of adherence to antihypertensive medications (19.1% vs 20.9% respectively) (Table 2).

**Table 2:** Behavioral and other determinants of hypertensive crisis among the respondents at selected public hospitals of eastern Ethiopia, 2024.

Variables	Category	Group of patients (N = 345)	
		Cases, n (%)	Controls, n (%)
Previous history of HTN	Yes	92(80.0)	131(57.0)
	No	23(20.0)	99(43.0)
Medication started	Yes	69(60.0)	124(53.9)
	No	9(7.8)	21(9.1)
Regular follow-up	Yes	41(35.7)	78(33.9)
	No	37(32.2)	67(29.1)
Family history of HTN	Yes	55(47.8)	109(47.4)
	No	60(52.2)	121(52.6)
Smoking cigarette	Yes	65(56.5)	87(37.8)
	No	50(43.5)	143(62.2)
Khat chewing	Yes	74(64.3)	159(69.1)
	No	41(35.7)	71(30.9)
Alcohol use	Yes	33(28.7)	26(11.3)
	No	82(71.3)	204(88.7)
Knowledge of HTN	Good	47(40.9)	140(60.9)
	Poor	68(59.1)	90(39.1)
Level of medication adherence	Low adherence	66(57.4)	113(49.1)
	Medium adherence	27(23.5)	69(30.0)
	High adherence	22(19.1)	48(20.9)

### Factors associated with hypertensive crisis

In a bivariate logistic regression model, 14 variables were significant at a p < 0.25. The explanatory variables were age, sex, employment, comorbidity, DM, stroke, coronary syndrome, kidney disease, asthma, migraine, history of HTN, smoking cigarette, alcohol drinking, and knowledge of HTN. However, multivariable logistic analysis identified six independent determinants of hypertensive crisis at p < 0.05: sex, employment, DM, previous history of HTN, alcohol drinking, and knowledge of HTN.

Females had three times higher odds of experiencing hypertensive crisis than males (AOR = 2.91; 95% CI 1.67, 5.07). Unemployed participants were 2 times higher to develop hypertensive crisis than individuals who had employment (AOR = 2.28; 95% CI 1.20, 4.33). The odds of having hypertensive crisis were 3 times higher among participants with DM than those without DM (AOR = 3.33; 95% CI 1.59, 7.01). Similarly, previous history of HTN were 2 times higher to develop hypertensive crisis (AOR = 2.25; 95% CI 1.21, 4.19).

The odds of having hypertensive crisis were also 3 times higher among individuals who have alcohol use than their counterparts (AOR = 3.01; 95% CI 1.45, 6.24). Additionally, respondents who had poor knowledge about HTN were 1.86 times (AOR = 1.86; 95% CI 1.07, 3.22) more likely to develop hypertensive crisis than individuals who had good knowledge of HTN (Table 3).

**Table 3:** Factors associated with hypertensive crisis among the respondents at selected public hospitals of eastern Ethiopia, 2024.

Variables	Category	Group of patients (N = 345)		COR (95% Cl)	AOR (95% Cl)	P-value
**Cases** **n (%)**	**Controls n (%)**			
Age category	18-45	38(33.0)	109(47.4)	1	1	
	46-65	53(46.1)	87(37.8)	1.75(1.06-2.89)	0.85(0.44-1.63)	0.620
	>65	20(20.9)	34(14.8)	2.03(1.07-3.84)	0.68(0.28-1.63)	0.388
Sex	Male	52(45.2)	155(67.4)	1	1	
	Female	63(54.8)	75(32.6)	2.50(1.58-3.96)	2.91(1.67-5.07)	0.000*
Employment	Unemployed	36(31.3)	43(18.7)	1.98(1.18-3.32)	2.28(1.20-4.33)	0.012*
	Employed	79(68.7)	187(81.3)	1	1	
Comorbidity	Yes	71(61.7)	108(47.0)	1.82(1.16-2.88)	0.81(0.38-1.73)	0.585
	No	44(38.3)	122(53.0)	1	1	
DM	Yes	43(37.4)	30(13.0)	3.98(2.32-6.82)	3.33(1.59-7.01)	0.001*
	No	72(62.6)	200(87.0)	1	1	
Stroke	Yes	29(25.2)	23(10.0)	3.04(1.66-5.54)	1.06(0.48-2.33)	0.886
	No	86(74.8)	207(90.0)	1	1	
Coronary syndrome	Yes	7(6.1)	6(2.6)	2.42(0.79-7.37)	2.40(0.64-9.04)	0.195
	No	108(93.9)	224(97.4)	1	1	
Kidney disease	Yes	24(20.9)	36(15.7)	1.42(0.80-2.52)	1.13(0.52-2.48)	0.760
	No	91(79.1)	194(84.5)	1	1	
Asthma	Yes	2(1.7)	15(6.5)	0.25(0.06-1.13)	0.22(0.04-1.11)	0.066
	No	113(98.3)	215(93.5)	1	1	
Migraine	Yes	4(3.5)	22(9.6)	0.34(0.12-1.01)	0.58(0.17-1.99)	0.382
	No	111(96.5)	208(90.4)	1	1	
Previous history of HTN	Yes	92(80.0)	131(57.0)	3.02(1.79-5.12)	2.25(1.21-4.19)	0.010*
	No	23(20.0)	99(43.0)	1	1	
Smoking cigarette	Yes	65(56.5)	87(37.8)	2.14(1.36-3.37)	1.61(0.92-2.82)	0.096
	No	50(43.5)	143(62.2)	1	1	
Alcohol used	Yes	33(28.7)	26(11.3)	3.16(1.78-5.61)	3.01(1.45-6.24)	0.003*
	No	82(71.3)	204(88.7)	1	1	
Knowledge of HTN	Good	47(40.9)	140(60.9)	1	1	
	Poor	68(59.1)	90(39.1)	2.25(1.43-3.55)	1.86(1.07-3.22)	0.028*

*Significant variable at a p-value < 0.05, HTN, Hypertension.

## Discussion

This study employed unmatched case control study, aims to assess the determinants of hypertensive crisis in public hospitals of eastern Ethiopia. In this study sex, employment, DM, previous history of HTN, alcohol drinking, and knowledge of HTN were found to be significant predictors of hypertensive crisis.

The findings of this study showed that female patients were 3 times higher risk of having hypertensive crisis than male patients. This finding is supported by a longitudinal study conducted in Switzerland [31], and a retrospective cross-sectional studies conducted in Ayder comprehensive specialized university hospital, Ethiopia [18]. The possible reason might be female patients were less aware of the cardiovascular problems associated with hypertension [32,33]. One possible reason might be high blood pressure in females can occur more frequently as a result of health conditions such as menopause, pregnancy, and birth control [34]. Women may not have enough access to healthcare due to cultural norms that prioritize male health over female health, financial constraints, or household responsibilities. Cultural dietary trends, such as excessive salt consumption or a lack of physical activity due to safety concerns or gender norms, might raise females blood pressure.

In the current study, unemployed participants were 2.3 times higher risk of having hypertensive crisis than among employed. This finding is in line with a hospital-based unmatched case control study conducted in the adult emergency departments of public hospitals in Addis Ababa, Ethiopia [35]. This might be due to unemployment can have emotional impacts such as worry, fear, and depression. These emotions can raise stress levels and increase the risk of cardiac problems and hypertension [36]. The other reason could be that those who were unemployed might not always be able to afford to acquire their medications, which could lead to a worsening of their clinical conditions. Unemployment can cause emotional detachment and social disengagement, which might worsen mental health and illness self-management. Due to their limited availability to nutritious foods, unemployed people may consume high-sodium, and low-nutrient diets, which might worsen blood pressure control.

This study revealed that the odds of having hypertensive crisis were 3 times higher among participants with DM than among those without DM. This is similar to a retrospective study conducted in Brazil [37], a case control study conducted in USA and Ethiopia [35,38]. This might be due to the fact that as a result of the hyperglycemic effect, individuals with diabetes mellitus have less vasodilator synthesis, and more pro-coagulant and vasoconstrictor release in their blood vessel walls, which contribute to atherosclerosis and raised blood pressure [39]. Compared to communicable diseases like HIV/AIDS, malaria, and tuberculosis, non-communicable disorders like hypertension in Ethiopia receive less funding and attention. In hypertensive patients, especially those with a history of DM, this lack of priority may lead to hypertensive crisis.

The findings of this study showed that participants who had previous history of HTN were 2.25 times higher odds of hypertensive crisis than those without a previous history of HTN. This finding is comparable with a prospective longitudinal study conducted in Switzerland, a systematic review and meta-analysis study in Africa, and unmatched case control study conducted in Addis Ababa, Ethiopia [35,40,41]. This might be due to the fact that, the risk of a hypertensive crisis is enhanced by a prior history of hypertension because persistently high blood pressure can cause ischemia and an increased workload for the heart [42]. The other reason could be in Ethiopia, religious healing and traditional remedies often take priority over medical treatments, leading to delays in diagnosis and medication adherence. Additionally, inconsistent patient record-keeping and the lack of electronic health systems hinder proper follow-up and continuity of care for hypertensive patients, especially in rural areas.

The results of this study showed that respondents who used alcohol had 3 times higher risk of experiencing a hypertensive crisis than those who did not. This finding is supported by a systematic review and meta-analysis study in Africa [41], and a prospective observational study conducted in India [43]. This might be because alcohol use can alter blood vessel muscles, which can narrow blood vessels and force the heart to pump blood throughout the body more forcefully, thus raising blood pressure dramatically [44].

This study also revealed that participants who had poor knowledge of HTN were 2 times higher odds of hypertensive crisis than those who had good knowledge of HTN. The possible reason might be associated with lack of understanding about the disease condition and overall management process and take care of their own negative side effects, which can result in uncontrolled hypertension.

### Strengths and limitations

One of the strength of this study is that it employed a case-control study design to assess the determinants of hypertensive crisis. The other strength of the study is the employment of a large sample size in four public hospitals, the use of multiple data collection techniques, including measurements, interviews, and reviews of medical charts, and the inclusion of several potential risk factors to determine their association with the onset of hypertensive crisis.

The limitation of this study is that the inherent nature of the case-control design, establishing a temporal relationship between risk factors and outcomes is challenging. Additionally, the study did not include patients from private hospitals or those who did not visit hospitals during the data collection period, which may limit the generalizability of the findings. Potential biases related to self-reporting, recall, and social desirability could also influence the results. Another limitation is the use of an unmatched case-control design, in which the control group was not matched by age, sex, or other sociodemographic factors. Given that aging is an established risk factor for complications from hypertension, including hypertensive crisis, the substantial age difference between the case and control groups may have created confounding effects. This might have affected the observed association between hypertension and hypertensive crisis by influencing other demographic and comorbidity factors. To overcome these confounding effects and guarantee consistent age distributions, future research could employ matching techniques or random selection methods.

### Conclusions

The results of this study show that, participants with DM, female sex, unemployment, alcohol drinking, past history of HTN, and poor knowledge of HTN were the independent determinants of HTN crisis. These results underline the necessity of focused public health initiatives, such as lifestyle modification programs, community-based hypertension education, and enhanced access to healthcare for vulnerable populations. The impact of Ethiopia’s hypertensive crisis could be lessened by incorporating these variables into national guidelines for managing hypertension. Additionally, implementing a risk-based triage system in emergency care settings would prioritize patients with diabetes mellitus, a history of hypertension, or alcohol consumption.

Both the community and the healthcare system should adopt focused efforts to lower the morbidity and mortality from HTN-crisis. Public awareness, early detection, and screening programs for hypertension should be strengthened by the government and health bureaus, with a focus on high-risk populations such as the unemployed and diabetics. In addition to improving follow-up treatment, healthcare workers should inform patients about modifiable risk factors such alcohol use and poor understanding of hypertension.

## Abbreviations

ACS: Acute Coronary Syndrome
AOR: Adjusted Odds Ratio
CHF: Congestive Heart Failure
CI: Confidence Interval
COR: Crude Odds Ratio
CSA: Central Statically Agency
DBP: Diastolic Blood Pressure
DM: Diabetes Mellitus
DRH: Dilchora Referral Hospital
ED: Emergency Department
HFCSH: Hiwot Fana Comprehensive Specialized Hospital
HGH: Haramaya General Hospital
HTN: Hypertension
JUSHYRH: Jigjiga University Sheik Hassan Yabare Referral Hospital
MmHg: Millimeter of mercury
